# New Treatments in Spinal Muscular Atrophy: Positive Results and New Challenges

**DOI:** 10.3390/jcm9072222

**Published:** 2020-07-13

**Authors:** Sonia Messina, Maria Sframeli

**Affiliations:** 1 Department of Clinical and Experimental Medicine, University of Messina, 98125 Messina, Italy; 2 NEuroMuscular Omnicentre (NEMO) Sud Clinical Centre, University Hospital “G. Martino”, 98125 Messina, Italy; mariasframeli@hotmail.it

**Keywords:** spinal muscular atrophy, therapy, nusinersen, risdiplam, onasemnogene abeparvovec

## Abstract

Spinal muscular atrophy (SMA) is one of the most common autosomal recessive diseases with progressive weakness of skeletal and respiratory muscles, leading to significant disability. The disorder is caused by mutations in the survival motor neuron 1 (*SMN1*) gene and a consequent decrease in the *SMN* protein leading to lower motor neuron degeneration. Recently, Food and Drug Administration (FDA) and European Medical Agency (EMA) approved the antisense oligonucleotide nusinersen, the first SMA disease-modifying treatment and gene replacement therapy by onasemnogene abeparvovec. Encouraging results from phase II and III clinical trials have raised hope that other therapeutic options will enter soon in clinical practice. However, the availability of effective approaches has raised up ethical, medical and financial issues that are routinely faced by the SMA community. This review covers the available data and the new challenges of SMA therapeutic strategies.

## 1. Introduction

Spinal muscular atrophy (SMA) is a heterogeneous hereditary neuromuscular disease, presenting with progressive weakness of skeletal and respiratory muscles, leading to muscle atrophy and significant disability. The disease is caused by a homozygous deletion or a heterozygous deletion combined with point mutation on the other allele on the survival motor neuron 1 (*SMN1*) gene on chromosome 5q and consequential lack of the *SMN* proteins, causing degeneration of lower motor neurons [1,2]. SMA encompasses a wide range of clinical severity and has been classified into subtypes according to age at onset and the maximum motor milestones achieved, ranging from the most severe SMA I to the mildest SMA IV.

SMA I patients never acquire the sitting position and present clinical onset before six months as “floppy infant” with reduced spontaneous movements and paradoxical breathing pattern. The life expectancy related to respiratory muscle failure is under two years without drug treatment [3]. SMA II shows a milder course with onset between six and 18 months. Patients are able to sit, but not walk independently and develop respiratory involvement that usually requires the use of non-invasive ventilation before adulthood and orthopedic complications such as severe scoliosis and joint contractures. SMA III and IV are the mildest forms with later onset, achievement of independent walking and variable clinical course, in SMA type IV usually without life-threatening events. SMA I and II patients often present failure to thrive and dysphagia and require to increase nutritional uptake with the use of hyperproteic and hypercaloric oral supplements or, in the more severe cases, gastrostomy placement. These patients require a multidisciplinary rehabilitative approach covering respiratory, orthopedic, psychological, physio- and speech-therapist and nutritional care [4,5].

SMA is one of the most common autosomal recessive diseases and causes of mortality in childhood with a carrier frequency of one in 40–67 adults and an incidence of one in 11,000 live births [6]. However, the epidemiologic burden of SMA differs among the subtypes. Several studies approached this issue showing a lower incidence for SMA III compared to the other forms. Ogino et al., in their review, proposed in SMA type I an incidence rate of 5.83 per 100,000 live births, in SMA type II 2.66 per 100,000 livebirths and in SMA type III 1.20 per 100,000 live births. Therefore, SMA type I, II and III constituted, respectively, 60%, 27% and 12% of all SMA cases [7]. A more recent review showed for SMA type I, II and III incidence rates of around 5.5, 1.9 and 1.7 per 100,000, respectively [8].

The clinical variability in SMA is mainly attributable to variable copy numbers of *SMN2*, which is a *SMN1* paralogous gene, producing a protein lacking exon 7 (*SMN*Δ7) due to alternative splicing, but also small amounts of functional *SMN* proteins. The number of *SMN2* copies is inversely related to clinical phenotypes [9]. However, several genetic modifiers, such as plastin-3 and neurocalcin delta, have been hypothesized to explain the clinical variability between patients with the same number of *SMN2* copies and within discordant families [10].

Recently, the antisense oligonucleotide (ASO) nusinersen and the gene replacement therapy by onasemnogene abeparvovec have become available for SMA patients. Moreover, encouraging results from phase II and III clinical trials have raised hope that other therapeutic options will enter soon in clinical practice [11,12,13,14,15]. However, the availability of effective approaches has raised up ethical, medical and financial issues that are routinely faced by the SMA community.

**Figure 1:**
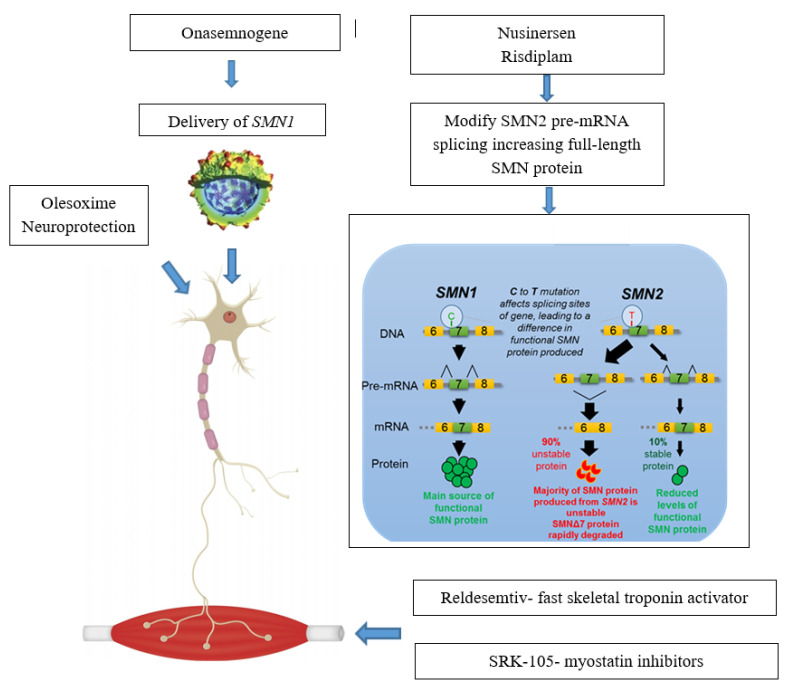
Main available therapeutic approaches and their mechanisms of action. *SMN1 =* survival motor neuron 1; *SMN2* = survival motor neuron 2; *SMN* = survival motor neuron.

Actual approaches can be subdivided in Survival Motor Neuron (*SMN*)—Dependent Gene Therapies, which act as splicing modificator of *SMN2* (nusinersen, small molecules) or replacing *SMN1* gene (onasemnogene abeparvovec) and in treatments targeting *SMN*—Independent Factors (muscle enhancing therapies and neuroprotection) (Figure 1).

This review covers the available data of SMA therapeutic strategies in pre-clinical development, currently tested in clinical trials and available in clinical practice.

## 2. *SMN*—Dependent Gene Therapies

### 2.1. Splicing Modification of SMN2

#### 2.1.1. Nusinersen

The first approved drug for SMA was nusinersen, which is an ASO that promotes the inclusion of exon 7 in mRNA transcripts of *SMN2*. Nusinersen binds to an intronic splice-silencing-site in intron 7 of *SMN2* and inhibits the action of other splice-factors, promoting exon 7 incorporation into the mRNA. This mechanism allows the translation of a higher level of fully functional *SMN* protein with a significant amelioration of survival and pathology in different SMA experimental models [16,17,18]. The journey of nusinersen towards approval and commercialization has been supported by several trials demonstrating efficacy without any major drug-related adverse event. ASOs do not cross the blood-brain barrier, therefore in all clinical trials nusinersen was administered intrathecally with a frequency of four times over two months in the initial loading period and every four months in the maintenance period.

After promising results for nusinersen in phase I and II trials in children with SMA type II and III [19,20], two phase III, randomized, double-blind, sham-procedure controlled studies were initiated consequently. ENDEAR (ClinicalTrials.gov identifier: NCT02193074, years 2014–2016) assessed safety and clinical efficacy of nusinersen in 121 infants with infantile-onset SMA and younger than seven months. In the interim analysis, infants treated with nusinersen had higher improvement in the motor milestone categories of the Hammersmith Infant Neurological Examination (HINE) than controls (41% vs. 0%, *p* < 0.001). Moreover, the nusinersen group demonstrated a prolonged time to death (hard ratio for death 0.37; *p* = 0.004) or need for permanent ventilation compared to controls and six out of 73 treated patients achieved independent sitting over a one year treatment period. Furthermore, infants with shorter disease duration at screening had better response to treatment [21].

CHERISH (ClinicalTrials.gov identifier: NCT02292537, years 2014–2017) involved 126 children with later-onset SMA. The median age at baseline was four years (two to nine years) in the treated group and three years (two to seven years) in the controls. Interim analysis after 15 months of treatment showed in the nusinersen group a mean increase of 4.0 points vs. a mean decrease of 1.9 points in controls (*p* < 0.001) in the Hammersmith Functional Motor Scale-Expanded (HFMSE) score. Regarding upper limb function, there was a least-squares mean increase from baseline to month 15 in the Revised Upper Limb Module (RULM) score in the nusinersen group of 4.2 points and a mean decrease in the control group of −0.5 points (*p* < 0.001). In the final analysis, 57% of nusinersen patients vs. 26% in the sham group had a rise of three points in HFMSE scores after 15 months of treatment [22]. Both trials were terminated prematurely on the basis of these positive results and patients continued treatment in an open-label extension study (SHINE, ClinicalTrials.gov identifier: NCT02594124, started in 2017 and ongoing) with the aim of assessing long-term effects of nusinersen in terms of safety and tolerability and clinical changes (HFMSE, RULM and WHO motor milestones). Interim analysis showed a continued benefit of nusinersen on HFMSE and RULM score over day 1170 of treatment and confirmed a more evident effect in younger patients at first dose (2.06 to <3.69 years) [23].

The ad interim results of the NURTURE open-label study (ClinicalTrials.gov identifier: NCT02386553, started in 2017 and ongoing) emphasized the importance of proactive treatment with nusinersen as soon as reached the genetic diagnosis in presymptomatic infants and strengthened the rationale for newborn screening (NBS). This study involved 25 presymptomatic SMA infants below six weeks of age and, after a median of 2.9 years of follow up, the effects of nusinersen were very encouraging. At a mean age of 34.8 (25.7–45.4) months of age, 100% of patients were alive, 92% achieved walking with assistance and 88% independently [24].

Nusinersen was approved by Food and Drug Administration (FDA) in December 2016 and by European Medical Agency (EMA) in June 2017. While waiting for regulatory approval, nusinersen was made available for compassionate use for SMA type I infants by the pharmaceutical company. The advent of an expanded access programs (EAP) using an intrathecal administration in fragile infants such as type 1 SMA has raised a number of issues, related to its planning and management, that have been dealt differently among countries [25]. However, “real-world data” confirmed therapeutic benefits with motor function improvements in SMA I [26,27,28,29]. We recently demonstrated in 85 SMA I patients (aged from two months to 15 years) after a year of treatment an amelioration on both the functional scales, Children’s Hospital of Philadelphia Infant Test of Neuromuscular Disorders (CHOP-INTEND) and HINE-2 for the whole group (*p* < 0.001) and the subgroups with two and three *SMN2* copies (*p* < 0.001). The difference was found not only in patients younger than 210 days at baseline (*p* < 0.001), but also on the CHOP-INTEND in those younger than five years and on the HINE-2 younger than two years [30].

#### 2.1.2. Small Molecules

Risdiplam, previously known as RO703406 and RG7916, is a small molecule that modulates *SMN2* gene splicing, binding two sites in *SMN2* pre-mRNA: 5′ splice site (5′ ss) of intron 7 and exonic splicing enhancer 2 (ESE2) in exon 7. The unique specificity of binding two sites increases levels of full-length *SMN* mRNA and protein, while reducing the impact on splicing of other pre-mRNA and avoiding the possibility of off-target effects [31]. Preclinical studies showed that risdiplam can reach the central nervous system and peripheral organs in vivo and can lead to a significant increase of *SMN* protein in blood, brain and muscles, with an increase survival in different SMA mouse models [32,33]. One of the advantage of this drug is the oral route of administration. While the intrathecal administration route of nusinersen mainly limits its effect to motoneurons of the central nervous system, the systemic distribution demonstrated in preclinical studies with risdiplam by oral administration allows to hypothesize a possible effect in other tissues. This is of relevance, as numerous studies in human and murine models indicate that SMA may actually be considered as a multi-system disorder with an involvement of neuromuscular junction, gastrointestinal-tract, cardio-vascular system, lung and liver [34,35].

A phase I study in healthy volunteers (ClinicalTrials.gov identifier: NCT02633709, years 2016 and 2017) identified the optimal dosage and demonstrated that risdiplam increases *SMN2*/*SMN*∆7 mRNA ratio in a dose-dependent manner [36].

Four ongoing phase II trials are assessing safety and efficacy of risdiplam in different SMA types. The results of these studies have been presented in several conferences but are not yet published. FIREFISH (ClinicalTrials.gov identifier: NCT02913482) is an open-label trial testing the effectiveness in SMA I patients between one and seven months of age with two copies of *SMN2*. Part 1 of FIREFISH has tested for a year the drug at two different dosages, while Part 2 assesses the effectiveness at the selected higher dose. Part 1 involved 21 patients: Four received the lower dose and 17 the higher dose that is currently being used in Part 2 of the trial. After receiving risdiplam for 12 months, 90.5% of infants were alive with no permanent ventilation, 33% in the whole cohort and 41% of infants treated with the higher dose were able to sit without support for at least 5 s and 86% of all infants showed a ≥4-point improvement in CHOP-INTEND score from baseline. Moreover, 94.7% of infants are able to feed orally or in combination with a feeding tube ad no infant has lost the ability to swallow [37]. At the cut-off point after 16 months of treatment, the median change from baseline in CHOP-INTEND score was 19 over 64 of maximum score and 5% of infants were able to stand supporting their weight and 10% were able to bounce [38]. Preliminary final results of the Part 2 have been recently presented. Event-free survival time was greatly improved in infants treated with risdiplam compared to natural history. Treated patients showed a significant improvement in HINE-2 and CHOP-INTEND after 12 months. Swallowing and feeding ability was maintained by the majority of infants. Nearly half of the treated patients did not require hospitalization up to 12 months [39].

SUNFISH (ClinicalTrials.gov identifier: NCT02908685, started in 2016 and open-label extension ongoing) is assessing safety, tolerability and effectiveness in SMA type II and III cases aged two to 25 years who are not ambulatory. Part 1 of SUNFISH (*n* = 51) evaluated safety, pharmacodynamics (PD), pharmacokinetic (PK) and optimal dosage of risdiplam, while its efficacy is tested vs. placebo with a 2:1 randomization in Part 2 (*n* = 180). The results of the SUNFISH Part II were presented very recently and the primary end-point was met with a least squares mean changes at the 32-item motor function measure from baseline significantly greater in patients receiving risdiplam vs. placebo (Δ = 1.55; *p* = 0.0156). Moreover, RULM total change from baseline was significantly greater in patients receiving risdiplam vs. placebo (Δ = 1.59; = 0.0028), whereas the difference was not significant at the HFMSE. At the SMA Independence Scale (SMAIS) caregivers and patients (≥12 years) in the treated group reported improvement in independence when completing activities of daily living [40].

JEWELFISH (ClinicalTrials.gov identifier: NCT03032172, started in 2018 and ongoing) is an open-label, exploratory study to assess the safety, tolerability, PD and PK in patients with a broad spectrum of age (six months–60 years), who have previously participated in a study with a therapy targeting *SMN2* pre-mRNA splicing.

RAINBOW FISH (ClinicalTrials.gov identifier: NCT03779334, started in 2019 and ongoing) is an open-label, single-arm, multicenter clinical study to investigate the efficacy, safety, PK and PD in pre-symptomatic infants enrolled between birth and six weeks of age.

Based on the above-mentioned positive results, risdiplam is under review for approval by FDA and a recent press release announced that FDA has extended the Prescription Drug User Fee Act date for its review of the New Drug Application of risdiplam till August 24 2020 [41]. In parallel, in many countries an EAP has been started for SMA type I patients (and in US also for SMA type II; ClinicalTrials.gov Identifier: NCT04256265), who were not eligible for treatment with currently approved treatments for SMA or cannot continue treatment as documented by the treating physician.

### 2.2. SMN1 Gene Replacement

Onasemnogene abeparvovec (previously known as AVXS-101) is a *SMN1* gene replacement therapy, which uses a non-replicating adeno-associated virus capsid (scAAV9) to efficiently deliver wild-type *SMN1* gene to motor neuron cells. This construct, an AAV9 vector carrying *SMN1* complementary recombinant DNA, can cross the brain–blood barrier, produces a sustained expression of *SMN* protein and prolongs survival of treated SMA-mice [42,43,44]. The main advantages of this approach are that a one-time injection is needed and it would lead to systemic expression of the *SMN* protein. Safety and tolerability has to be strictly monitored as acute hepatotoxicity and sensory neuron toxicity were reported in primates and piglets following high-dose intravenous administration of AAV vectors expressing human *SMN* [45]. Another issue could be the reported presence of pre-existing anti-AAV9 antibody in the SMA population [46].

AVXS-101-CL-101 (ClinicalTrials.gov identifier: NCT02122952, years 2014–2017) is an open-label study of 15 SMA type I cases with bi-allelic *SMN1* mutations (deletion or point mutations) and two copies of *SMN2*. Onasemnogene abeparvovec was administered as a single intravenous injection at two different doses: Twelve patients receiving 2.0 × 10^14^ vector genomes (vg) per kg and three receiving 6.7 × 10^13^ vg per kg. Serum aminotransferase levels increased in four cases, but returned to normal levels after treatment with corticosteroids. After the elevation in the first patient, the protocol was amended with addition of oral prednisolone for four weeks after drug administration. In the high-dose group, the scores of the CHOP-INTEND showed a rapid increase of 9.8 points after one month and 15.4 points after three months, compared to the decrease observed in SMA type I natural history [47]. Recently, a comparison with SMA type I untreated patients of the NeuroNEXT (NN101) study (ClinicalTrials.gov identifier: NCT01736553, started in 2017) [48] showed that after 24 months of follow-up the survival rate was 100% in AVXS-101-treated infants and 38% in the NN101 study cohort. Baseline mean CHOP-INTEND score was 28.2, improving to 56.5 in the treated group, compared to 20.3 with a decrease to 5.3 in controls. Moreover, 11 (92%) of the AVXS-101–treated infants were able to sit unassisted for ≥5 s, 10 (83%) for ≥10 s, nine (75%) for ≥30 s and two (17%) could stand and walk independently. CHOP-INTEND scores suggested that patients in the NN101 cohort did not achieve any motor milestones [49]. Further analysis demonstrated that the best predictors of functional amelioration were age below three months and high CHOP-INTEND scores at baseline [50].

The results of the STR1VE study (ClinicalTrials.gov Identifier: NCT03306277, 2018–2020) have been recently presented [51]. STR1VE-US is a part of the global phase 3 STR1VE clinical program. This includes open-label, phase 3, single-arm, single-dose, multi-center trials (STR1VE-US in the United States, STR1VE-AP in Asia Pacific and STR1VE-EU in Europe) designed to assess safety and efficacy in symptomatic patients with SMA type I < six months of age with one or two copies of the *SMN2* gene. In STR1VE-US, 20 of 22 patients (91%) met the co-primary efficacy endpoint of event-free survival at 14 months and 13 (59%) met the co-primary efficacy endpoint of functional sitting for ≥30 s at 18 months of age. Thirteen patients (59%) could sit independently for ≥30 s (*p* < 0.0001 vs. natural history) at the 18 months of age. Fifteen patients (68.2%) remain free of non-invasive ventilatory support during the study. Eighteen (81.8%) were free of ventilatory support at 18 months of age. CHOP-INTEND scores ameliorated by a mean of 6.9 points at one month, 11.7 points at three months and 14.6 points at six months after treatment. Twenty-one patients (95%) reached a CHOP-INTEND score ≥40 and 14 (64%) a score ≥50.

SPR1NT (ClinicalTrials.gov Identifier: NCT03505099, started in 2018) is an ongoing Phase 3, open-label, single-arm, multi-center trial designed to assess safety and efficacy of a one-time intravenous infusion in presymptomatic patients with SMA below six weeks of age and two or three copies of *SMN2.* Fourteen patients with two copies and 15 patients with three copies of *SMN2* were treated.

The completed clinical trial, START (ClinicalTrials.gov Identifier: NCT02122952, 2018–2020), enrolled 15 patients with infantile-onset SMA, 12 in a high-dose and three in a low-dose cohort. By 24 months following infusion, none of the patients the high-dose cohort required permanent ventilation. Patients in the low-dose cohort did not reach the ability to sit without support; in the high-dose cohort, nine of 12 patients (75%) reached the ability to sit without support for ≥ 30 s and two patients (17%) to stand and walk independently. These results showed a dose-response relationship.

The longer follow-up data were provided from the START Long-Term Follow-Up (LTFU) (ClinicalTrials.gov Identifier: NCT03421977). This is an ongoing, observational, long-term follow-up study of patients who completed START and electively enrolled in the study. The mean age of patients was 4.8 years (range 4.3–5.6 years) and the mean time since gene therapy treatment was 4.5 years (range 4.1–5.2 years). Of the 10 patients from cohort 2 (high dose of START) who enrolled in LTFU, all are free of permanent ventilation. No changes in the previously achieved milestones have been reported during the follow up period. Two patients have reached the ability of standing with assistance (neither of whom have received treatment with nusinersen) during the follow up period.

Based on these affirmative results, in May 2019 FDA approved onasemnogene abeparvovec for the treatment of SMA patients with less than two years of age with bi-allelic mutations in the *SMN1* gene, including those who are pre-symptomatic at diagnosis [52]. Furthermore, in May 2020 the European Commission (EC) granted conditional approval for onasemnogene abeparvovec for SMA type I patients with a bi-allelic mutation in the *SMN1* gene or for SMA patients with a bi-allelic mutation in the *SMN1* gene and up to three copies of the *SMN2* gene. In both cases, the approval covers SMA patients with a weight up to 21 kg according to the dosing guidance [53].

## 3. Treatments Targeting Survival Motor Neuron (*SMN*)—Independent Factors

### 3.1. Muscle Enhancing Therapies

Reldesemtiv (previously known as Tirasemtiv and CK-2127107) is a selective small-molecule troponin activator in fast skeletal muscles. The rationale for its use in SMA stands on several lines of evidence. This molecule increases the affinity of troponin C to calcium, sensitizes the sarcomere to calcium effects and reinforces contraction [54,55]. Moreover, Reldesemtiv has also been demonstrated to promote muscle response to nervous stimulus in humans [56]. Following a phase I study confirming its safety, a phase II, double-blind, randomized, placebo-controlled trial (ClinicalTrials.gov identifier: NCT02644668, years 2015–2018) on 70 patients with SMA type II to IV examined its effect on functional and respiratory performances. The compound were administered orally at two different doses (150 mg × 2/day and 450 mg × 2/day). The results showed, in the higher dosage group, a trend towards an increase from baseline in the six-minute walk test (6MWT) and of the maximal expiratory pressure (MEP). Adverse events were similar between treated and placebo groups [57].

SRK-015 is a monoclonal antibody, which selectively inhibits myostatin, promoting muscle cells growth and differentiation and improving muscle force in SMA mice [58,59]. A phase I trial (ClinicalTrials.gov identifier: NCT02644777, years 2017–2018) confirmed its safety and tolerability. A phase II study (TOPAZ, ClinicalTrials.gov identifier: NCT03921528, started in 2019 and ongoing), involved 58 SMA type II and SMA III patients, aged two to 21 years. Patients have received treatment by intravenous infusion every four weeks for one year. The six-month interim results will be available by the end of 2020.

### 3.2. Future Prospectives in SMN Independent Therapeutic Targets

Several *SMN* independent factors have been identified over the last years as involved in SMA pathogenesis on the basis of in vitro and in vivo studies and therefore they could represent future therapeutic targets.

Autophagy is the process by which cytoplasmic contents are delivered by autophagosomes to the lysosomes for degradation. In vitro and in vivo studies reported an increase of autophagosomes in the cytoplasm of SMA motoneurons, suggesting that autophagy dysregulation might alter intracellular trafficking, leading to cytotoxicity [60,61,62]. Moreover, intramuscular injections of the neurotrophic factor tetanus toxin heavy chain (TTC) can reduce the expression of autophagy markers (Becn1, Atg5, Lc3, and p62) in *SMN*Δ7 mice muscles without effects on weight and survival time [63]. Furthermore, inhibition of autophagy by intracerebroventricular administration of 3-methyladenine (3-MA) has been shown to ameliorate autophagic features, increase lifespan and improve motor performances in SMA pups [62].

Autophagy and apoptosis are linked in SMA as shown by the fact that 3-MA administration reduces also apoptotic cell death in the lumbar spinal cord [62]. Apoptosis has been demonstrated to be involved in SMA pathogenesis by several evidence. *SMN* protein decrement promotes apoptosis in vitro [64]. The c-Jun NH2-terminal kinase (JNK) cascades, known to have a pro-apoptotic role, is activated in *SMN*Δ7 mice and in SMA patients [65]. Moreover, the double JNK3-SMNΔ7 knockout (KO) mouse model shows a milder SMA phenotype [65]. Furthermore, JNK pharmacological inhibition ameliorates morphological features, improves motor performances and lifespan of SMA mice [66].

Another possible therapeutic target is agrin, a synaptic organizer relevant for the efficiency of neuromuscular transmission. A reduction of 50% in agrin expression levels has been found in muscle of *SMN*Δ7 mice. The administration of C-terminal fragment of mouse agrin to SMA pups can restore the crosstalk between muscles and motoneurons with positive effects on the maturation of the neuromuscular junction and on muscle tropism [67]. The repletion of agrin parallels to an amelioration on the overall disease phenotype and to a prolonged survival in severely affected SMA model mice [68].

Interestingly, the nuclear factor-kappa B pathway, deeply studied in another neuromuscular disorder in childhood, such as Duchenne muscular dystrophy, seems to be implicated also in SMA pathogenesis. This pathway modulates cell survival in mice spinal cord motor neurons induced by neurotrophic factors and its inhibition causes *SMN* reduction in SMA motoneurons [69,70,71].

Therefore, in conclusion, the results of these studies are promising, but additional evidence is needed before clinical translation of new compounds acting on these cellular/molecular pathways.

## 4. Discussion

The management of SMA is deeply changing and several issues have to be considered as clinicians use these innovative, effective and expensive new treatments (Table 1).

Regardless of the approach, the presented studies demonstrated better efficacy in SMA children pre-symptomatic or with the shortest disease duration [72]. This is a consequence of the rapid denervation process occurring in the first six months of life and the aim of pre-symptomatic treatment is the precocious rescue of motoneurons. However, we recently demonstrated that the mean age at diagnosis is 4.70 months (SD ± 2.82) in SMA type I, 15.6 months (SD ± 5.88) in SMA type II, and 4.34 years (SD ± 4.01) in SMA type III [73]. Therefore, these findings support the need of NBS to achieve a better efficacy of the available therapeutic options. NBS is nowadays performed in Italy, Taiwan, some States in US, Belgium and Germany as pilot studies [74,75,76,77,78]. The decision of how to handle newborns who test positive in the screening is crucial. An algorithm, based upon *SMN2* copy number, has been proposed by the SMA NBS Multidisciplinary working group, sponsored by CureSMA. The experts reached a consensus to start immediately treatment in infants with one, two and three *SMN2* copies, regardless of the presence of symptoms and to strictly follow patients with four copies till symptoms’ onset and then start treatment. However, it is still under debate how to handle families and infants with four or more copies and expected to have mild and late-onset disease [79,80,81]. Moreover, this aspect is further complicated by the fact that the correlation between *SMN2* copies and phenotype can be influenced by genetic modifiers, as demonstrated in siblings with the same SMA genotype [82]. A qualified genetic counselling and psychological support are mandatory to help parents to face the stressful situation to receive a severe diagnosis in an apparently healthy baby and to take relevant treatment decisions.

The variability in treatment response among patients could be better understood with the availability of reliable biomarkers. This knowledge would help to identify prognostic factors, to avoid long-term exposure to expensive drugs with still unknown long-term drug-related adverse events. A variety of biomarkers is currently under investigation including epigenetic, genetic, proteomic, electrophysiological and imaging tools [83]. Neurofilaments (NFs) muscle-specific miRNAs (myomiRs) and CSF proteomic profile, although biomarkers are not yet validated, have recently drawn attention as promising tools in SMA. NFs are markers of axonal degeneration. In infants with SMA type I the level of CSF NFs was significantly higher than in controls, with a response to nusinersen treatment that correlated with clinical improvement [84]. Similar results were confirmed in plasma in the ENDEAR study in symptomatic SMA type I patients [85]. In older patients with less severe forms of SMA, the role of NFs has not yet been confirmed probably as consequence of a slower disease progression [86,87]. *SMN* protein regulates RNA metabolism and biogenesis of microRNA (miRNA), which are gene expression modulators, and their dysregulation is implicated in a variety of neuromuscular diseases. Recently, a reduced expression level of circulating myomiRs miR-133a, -133b, miR-206 and −1 has been demonstrated in SMA type II and III patients under nusinersen treatment. Moreover, miR-133a decrement could be a predictor of motor function response to therapy [88]. A recent study evaluated, by mass spectrometry, non-targeted CSF proteomic profiles in SMA type II and III patients. The analysis highlighted two groups with differences in age and expression of proteins related to neurodegeneration and neuroregeneration. Moreover, intraindividual CSF differences were present between non-responders and responders to nusinersen treatment, with a correlation with motor functional improvement in the latter group [89].Considering the high costs of the approved new treatments and the limited data on long-term efficacy and safety, the scientific community feels the burning need to systematically collect “real-world data” to provide evidence for clinical decision-making and reimbursement. Only one study reported the results of a number needed to treat (NNT) analysis comparing the efficacy of nusinersen and onasemnogene abeparvovec on several outcomes using data from AVXS-101-CL-101 and ENDEAR studies in symptomatic SMA type I infants. Authors demonstrated an efficacy advantage of onasemnogene abeparvosec in terms of motor milestones achieved, motor function (CHOP-INTEND score) improvement and independence from permanent assisted ventilation. Moreover, the probability of preventing death was 20% higher in the onasemnogene abeparvosec treated group [90]. However, the main drawback of this study is the use of an unanchored indirect analysis of NNT between two studies. Head-to-head clinical trials should be performed to estimate comparative efficacy of the available approaches, avoiding possible biases such as differences in study design and patient characteristics. Moreover, a recent study highlights wide variations in cost and benefit estimates of nusinersen and indicates that onasemnogene abeparvosec is unlikely to represent “value for money” according to current standards of reimbursement for the UK NHS [91]. On the contrary, a US study demonstrated an incremental cost-effectiveness ratio (ICER), expressed as cost/quality-adjusted life year ($/QALY), of $46,947 for chronic treatment with nusinersen and of $31,379 base case at a price of $5M for onasemnogene abeparvosec, indicating that the latter was cost-effective with prices of ≤$5M [92]. Several international disease-specific registries have now been established (e.g., the International SMA Consortium Spinal Muscular Atrophy Patient Registry (iSMAC), the TREAT-NMD registry and the SMArtCARE project), collecting “real-world data” on treated and untreated SMA patients with standardized outcome measures, including patient-oriented tools on a longitudinal setting [93,94,95,96,97,98].

All studies so far have been conducted in patients followed in tertiary referral centers with the recommended standards of care and this likely contributed to the positive results of the trials. Nowadays, families have different therapeutic options, but it has to be highlighted by clinicians that the efficacy of these new compounds is significantly related to the adherence to a careful multidisciplinary management of care. Moreover, increment in muscle strength, acquisition of new milestones and increased survival are bringing attention to emerging phenotypes [15]. Treated SMA type I patients can be able to sit also unsupported and; therefore, with the effect of gravity, they exhibit a higher rate of scoliosis with often severe kyphoscoliosis in the first years of life. A careful X-ray and clinical monitoring, the use of braces and eventually the surgical option with “growing rods” are crucial. Moreover, the increase in muscle strength promotes the worsening of contractures, therefore intensive stretching and the use of standing frame or knee-ankle-foot-orthoses when possible have to be envisaged. The reported cognitive involvement might be more frequent in longer-surviving SMA type I patients and this aspect has to be elucidated in long term follow-up [99].

## 5. Conclusions

We are facing an exciting era with three available therapeutic options in a disease considered incurable for more than a century. NBS leading to early treatment is vital to provide optimal care. Each therapeutic strategy has its weaknesses and strengths and clinicians need to know them to optimize clinical care. Once these approaches will be available for all SMA types, the final choice will be based on patient’s clinical features and compliance and on the feasibility of drug administration. Since post-symptomatically treated patients are not cured, patients and parents ask for combined approaches. Additional therapies to complement present and forthcoming *SMN*-targeted treatments are needed in order to optimize their effects. Combinations of different drugs that increase *SMN* level or with muscle enhancing therapies must be tested in clinical trials.

The validation of prognostic factors and biomarkers to precisely detect evidence of response would decrement the time of exposure to expensive medications with unknown long-term drug-related adverse events, identifying in non-responders accurate treatment discontinuation criteria. The complementary effort by patients, care-givers, SMA advocacy groups and policymakers is required to continue promoting innovation for the benefit of SMA patients.

## Figures and Tables

**Table 1:** Main clinical developments in spinal muscular atrophy (SMA).

Approach /Compound	Sponsor	Mechanism	Trials’ Phase (SMA Type)	Administration	FDA Approval
Splicing modifiers of *SMN2* gene					
Nusinersen	Ionis-Biogen	ASO	I, II and III (I, II, III)	Intrathecal	X
Risdiplam	Roche	Small molecule	I, II and III (I, II, III)	Oral	pending
Albuterol		Beta-adrenergic agonist	Off-label	Oral	
Replacing *SMN1* gene					
Onasemnogene abeparvosec	Novartis-Avexis	AAV-9-vector construct	I, II and III (I, II)	Intravenous	X
Onasemnogene abeparvosec	Novartis-Avexis	AAV-9-vector construct	I	Intrathecal	
Muscle enhancing					
Reldesemtiv	Cytokinetics	Troponin activator	I and II (II, III, IV)	Oral	
SRK-105	Scholar Rock	Myostatin inhibitor	I and II (II, III)	Intravenous	
Neuroprotection					
Olesoxime	Hoffmann-La Roche	Anti-apoptotic agent	I and II (II, III) (development ended in 2018)	Oral	

ASO = antisense-oligonucleotide; AAV = adeno-associated virus; FDA= Food and Drug Administration.
